# Development of super nanoantimicrobials combining AgCl, tetracycline and benzalkonium chloride

**DOI:** 10.1186/s11671-024-04043-3

**Published:** 2024-06-11

**Authors:** Syed Imdadul Hossain, Diellza Bajrami, Nazan Altun, Margherita Izzi, Cosima Damiana Calvano, Maria Chiara Sportelli, Luigi Gentile, Rosaria Anna Picca, Pelayo Gonzalez, Boris Mizaikoff, Nicola Cioffi

**Affiliations:** 1 Chemistry Department, University of Bari Aldo Moro, Via E. Orabona, 4, 70126 Bari, Italy; 2 CSGI (Center for Colloid and Surface Science) c/o Dept. Chemistry, Via E. Orabona, 4, 70126 Bari, Italy; 3 Institute of Analytical and Bioanalytical Chemistry, Ulm University, Albert Einstein-Allee 11, 89081 Ulm, Germany; 4 ASINCAR (Research Association of Meat Industries of Principado de Asturias), 33180 Noreña, Spain; 5 Hahn-Schickard, Sedanstrasse 14, 89077 Ulm, Germany

**Keywords:** AgCl, Nanoparticles, Synergistic antimicrobials, Antibiotic, Bioactive surfactant, Biofilm

## Abstract

In this work, we demonstrate that a simple argentometric titration is a scalable, fast, green and robust approach for producing AgCl/antibiotic hybrid antimicrobial materials. We titrated AgNO_3_ into tetracycline hydrochloride (TCH) aqueous solution, thus forming AgCl/TCH in a one-step procedure. Furthermore, we investigated the one-pot synthesis of triply synergistic super-nanoantimicrobials, combining an inorganic source of Ag^+^ ions (AgCl), a disinfecting agent (benzyl-dimethyl-hexadecyl-ammonium chloride, BAC) and a molecular antibiotic (tetracycline hydrochloride, TCH). Conventional antimicrobial tests, industrial biofilm detection protocols, and in situ IR-ATR microbial biofilm monitoring, have been adapted to understand the performance of the synthesized super-nanoantimicrobial. The resulting hybrid AgCl/BAC/TCH nanoantimicrobials are found to be synergistically active in eradicating *Salmonella enterica* and *Lentilactobacillus parabuchneri* bacteria and biofilms. This study paves the way for the development of a new class of super-efficient nanoantimicrobials that combine relatively low amounts of multiple active species into a single (nano)formulation, thus preventing the development of antimicrobial resistance towards a single active principle.

**Supplementary Information:**

The online version contains supplementary material available at 10.1186/s11671-024-04043-3.

## Introduction

The development of an easy and green route towards synergistic silver halide/QAC (quaternary ammonium compound) colloids play a vital role in fighting one of the top 10 public health threats, e.g., the so called antimicrobial resistance (AMR) [1–4]. QACs are the major class of cationic surfactants, which contain at least one hydrophobic hydrocarbon chain linked to a positively charged nitrogen atom, and other alkyl groups which are mostly short-chain substituents such as methyl or benzyl groups. Although there is not a univocal interpretation of silver-based nanoparticles (NPs) biocidal action yet, it is thought that one of their main mechanisms is based on ion release in solution [5].

Nanotoxicological issues are limiting the applications of ultrafine metal NPs. In fact, it is well known that small (i.e., below 20 nm) metal NPs might penetrate into tissues, biological barriers, and cellular membranes [6, 7] causing adverse toxic effects in human organism. Hypothesized pathways of NPs uptake in the human body and target organs (where they can accumulate) rely on skin and nasal/pharyngeal mucosa penetration. From there, through the central cardiovascular system, NPs can easily reach organs [8, 9]. Toxicity of NPs varies with their properties (size, shape, charge, surface energy, chemical composition and others) [10, 11] and it depends on the properties of living organisms [12]. Alternative materials offering a controlled release of bioactive metal ions, can be coordination polymers, ionic-exchangers, and particles composed by insoluble salts. Particularly, the use of intrinsically insoluble AgCl in place of elemental Ag could increase the level of control over the extent and rate of bioactive species generation, by providing a thermodynamically-controlled release of Ag^+^ ions [1, 2, 13]. AgCl NPs have already been applied in biomedical, cosmetic, and food packaging sectors [14, 15]. Previously, preparation and characterization of AgBr nanocolloids were presented by Panda et al. where a quaternary ammonium compound acts as both the source of bromide ions and the stabilizing agent [16]*.* Recently, a standard argentometric titration was found to be a scalable, facile, versatile, fast, and robust technique for the production of AgCl-based antimicrobial agents [1]. AgCl/BAC was prepared by titrating an aqueous solution of AgNO_3_ into an aqueous benzyl-dimethyl-hexadecyl-ammonium chloride (BAC) solution. The possible use of a wide range of solvents and QACs makes the system extremely flexible; a peristaltic pump can be used to drop the silver precursor, if a large-scale production is required [17]. In the present work, combining silver chloride nanoparticles with TCH antibiotic for biofilm inhibition and bacteria treatment presents several novel aspects in the field of antimicrobial application: synergistic triply antimicrobial effect, biofilm disruption, possible reduction of antibiotic resistance, follow green and easy approach, extended stability and shelf-life of TCH antibiotic, and broad-spectrum activity against both Gram-positive and Gram-negative strains. The synergy allows for lower concentrations of both the nanoparticles and antibiotics to be effective, reducing potential toxicity while maintaining efficacy against bacteria [18–20]. These properties make this scalable approach promising for combating bacterial infections, particularly those associated with biofilm formation and antibiotic resistance.

Indeed, the involvement of Cl-containing antibiotics, such as tetracycline hydrochloride, clindamycin hydrochloride, and chlortetracycline hydrochloride, can be a turning point of synergistic systems, which can be more lethal against food pathogenic bacteria consortia, i.e., biofilms. Tetracycline hydrochloride (TCH) is a group of broad-spectrum antibiotics. The main mechanism of action for Tetracycline is the inhibition of bacterial protein synthesis. The strong binding of tetracycline to the bacterial 30S ribosomal subunit leads to the inhibition of protein synthesis by causing the rupture of codon–anticodon interactions between tRNA and mRNA. This results in the bond interruption between the aminoacyl-tRNA and the ribosomal acceptor site [21]*.* Several studies have been conducted on the ex-situ or in-situ preparation of tetracycline-combined AgNPs as antimicrobial agents [22–25]. Nonetheless, those studies are limited to lab-scale production, and may raise (nano)toxicity issues due to the presence of toxic solvents and reducing agents. Additionally, they usually require complex preparation routes and lead to NPs of uncontrolled size and shape. Furthermore, the use of a larger amount of antibiotic addition in the nanocomposite could lead to a loss of environmental and economic sustainability.

*Salmonella genus* is a major pathogen which is responsible for one of the most common and dangerous foodborne disease called Salmonellosis [26]. It has ability to form biofilm, which cause high resistance against disinfection and antibiotics. Heterofermentative lactic acid bacteria like *L. parabuchneri* are usually responsible for contaminating dairy products and increasing food toxicity level due to the histamine release. *Lentilactobacillus parabuchneri* strains produce biofilms on the surface of dairy industry equipment and become resistant to disinfecting agents while act as reservoirs of histamine-producing bacteria [27, 28]. Therefore, there is an acknowledged need for innovative antimicrobials against them to fight the antibiotic resistance and prevent biofilm formation [29–31].

In the present study, 10 mM AgCl/BAC/TCH colloids were developed following a simple titration method that has been adapted to the production of a multicomponent super-antimicrobial hybrid material. A 9:1 ratio was used between BAC and TCH. BAC is already known to be a bioactive and stabilizing material, and the addition of a very small concentration of TCH (1 mM), surprisingly made the system highly active in eradicating *S. enterica* biofilms. The AgCl/BAC/TCH nanoparticles can be easily embedded in different polymeric matrices, thus resulting in effective antimicrobial surfaces, as demonstrated by the in-situ IR monitoring of *L. parabuchneri* biofilm inhibition on AgCl/BAC/TCH/PVMK coatings.

## Material and methods

### Materials

Silver nitrate (AgNO_3_), BAC, and Poly(vinyl methyl ketone), acetonitrile LC–MS grade, trifluoroacetic acid (TFA), 2,5-di-hydroxy-benzoic acid (DHB), 9-amminoacridine (9-AA), α-cyano-4-hydroxy-cinnamic acid (CHCA) were purchased from Sigma–Aldrich (Milan, Italy). Tetracycline hydrochloride TCH (480.9 g/mol; ≥ 95%) was purchased from Merck (Darmstadt, Germany). Milli-Q water and isopropyl alcohol (IPA, anhydrous, 99.5%, Sigma Aldrich, Milan, Italy) were used throughout the experiments.

### Preparation of AgCl/BAC/TCH colloids

First, stock solutions of 10 mM AgNO_3_, 10 mM BAC, 10 mM and 1 mM TCH were prepared in Milli-Q water. The synthesis protocol for AgCl/BAC NPs has been previously published [1]. Briefly, 10 mL of AgNO_3_ (10 mM) titrating solution was added to 10 mL of BAC (10 mM) for the production of 10 mM AgCl/BAC aqueous solutions. Similarly, 10 mM AgCl/NaCl was prepared by adding AgNO_3_ to 10 mM NaCl in aqueous medium. For AgCl/BAC/TCH, 10 mL of AgNO_3_ (10 mM) titrating solution were added to a mixture of 9 mL of BAC (10 mM) and 1 mL of TCH (10 mM) (BAC:TCH ratio 9:1). A similar approach was adopted for the production of the AgCl/NaCl/TCH NPs (NaCl:TCH ratio 9:1).

### TEM and DLS characterization

TEM was performed using a FEI Tecnai 12 instrument (120 kV; filament: LaB_6_). The AgCl/BAC/TCH and AgCl/TCH NPs were drop-cast onto copper grids (300 mesh, Agar Scientific) in volumes of 2–3 μL for each sample. Dynamic light scattering (DLS) measurements were performed using a Zetasizer Nano ZS instrument (Malvern Instruments, Ltd., Worcestershire, UK). The Zetasizer Nano ZS is equipped with a 4 mW He−Ne laser and an automatic laser attenuator, and with an avalanche photodiode detector. All measurements were recorded at the scattering angle θ = of 173°; ζ-potential measurements at θ = 12.8°. The temperature was set to 25 °C. The hydrodynamic radius (RH) was determined using the Stokes−Einstein equation.

### MALDI MS characterization

The AgCl/BAC/TCH and AgCl/TCH NPs were pelleted by ultracentrifugation at 14,000 rpm for 30 min. The obtained pellets were redissolved in 150 µL of pure water; as a control a 1 mM solution of TCH was also prepared in water. For MALDI analyses, 5 μL of the resulting solution were mixed with an equal volume of each matrix solution (CHCA, DHB, 9AA 10 mg/mL in 70:30 ACN:0.1% TFA); 1 μL of this solution was then spotted onto a MALDI plate and allowed to dry. Unless otherwise specified the dried-droplet method was thoroughly used in this work. Analyses also in LDI modality were carried out. All experiments were performed using a 5800 MALDI ToF/ToF analyzer (AB SCIEX, Darmstadt, Germany) equipped with a neodymium-doped yttrium lithium fluoride (Nd:YLF) laser (345 nm), in reflector positive or negative ion mode with a typical mass accuracy of 5 ppm. In MS and MS/MS mode 1000 laser shots were normally accumulated by a random raster pattern, at laser pulse rate of 400 and 1000 Hz, respectively; mass spectra shown in the following were averaged on at least five single mass spectra (1000 laser shots each). MS/MS experiments were performed setting a potential difference of 1 kV between the source and the collision cell; collision-induced dissociation (CID) modality was activated using argon as the collision gas with a medium pressure of 10^−6^ Torr. The delayed extraction (DE) time was set at 400 ns. The laser fluences used were fixed close to laser threshold for each matrix within a range of 1.9–2.5 J/m^2^. DataExplorer software 4.0 (AB Sciex) was used to control the acquisitions and to perform the initial elaboration of data while SigmaPlot 11.0 was used to graph final mass spectra.

### Bacterial strains and culture conditions

Gram negative bacteria *S. enterica* CECT4594, obtained from the Spanish Type Culture Collection (CECT) of University of Valencia, was prepared with tryptic soy broth (TSB, Oxoid, the UK) at conditions instructed by the provider. It was stored with 20% glycerol (in TSB) at − 80 °C. One of the stocks cryovial was thawed and plated on tryptic soy agar (TSA, Oxoid, the UK). The plates were incubated at 37 °C for 24 h and then stored in fridge at 4 °C. At each use, bacterial suspensions were prepared by isolated single colonies taken from these plates, and incubated overnight in Mueller Hinton Broth (MHB, Sigma Aldrich, the USA) at 37 °C. The final concentration of the cultures was adjusted to 0.5 OD (optical density) in MHB at 500 nm, by a UV–Vis spectrophotometer (Biochrom Libra S60), reaching to 10^8^ CFU/mL.

The Gram-positive bacterium *L. parabuchneri* DSMZ5987 was obtained from the Leibniz Institute, German Collection of Microorganisms and Cell Cultures (DSMZ), Niedersachsen, Germany. Bacterial cells were maintained in De Man, Rogosa and Sharpe (MRS) broth in a microaerophilic environment during incubation at 37 °C for 24 h. At the end of the exponential growth phase, the OD_600_ of the bacterial solutions was monitored using a UV–Vis spectrophotometer (Specord S600, Analytik Jena AG, Germany). Cells were resuspended in fresh MRS medium to reach OD_600_ = 0.7 ready for in situ IR spectroscopy [32]. The strain was isolated at − 80 °C in MRS with 10%_*w*/*v*_ sterile glycerol.

### Antimicrobial and antibiofilm protocols

The agar well diffusion method [33] was used to study NPs antimicrobial activity. *Salmonella enterica* bacterial culture was serially diluted to a final concentration of 10^6^ CFU/mL. 1 mL of this bacterial suspension was mixed with 20 mL of TSA solution and spread into Petri dishes. A 2 mm hole was created in the agar plate with autoclaved slender tubes and filled with 20 µL of either antimicrobial or control (sterilized Milli-Q water) solutions. Then, those plates were incubated at 37 °C for 24 h, to measure the inhibition zones.

The viability assays by determination of CFU counting were conducted prior to real-time infrared studies. The capability of the AgCl nanoantimicrobials as potential materials to inhibit biofilms has been tested via viable cell standard quantification method for separating individual cells on an agar plate by growing colonies of bacterial cells, consequently differentiating dead cells from living biomass and quantifying vital cells [17, 34]. Experimental details can be seen in supplementary information.

The biofilm formation protocol for HDPE can be found elsewhere [35]. Briefly, *S. enterica* bacterial strain was used for the formation of biofilms on HDPE (3 × 2 cm^2^) coupons. The HDPE coupons were sterilized prior to the experiments using 1000 ppm peracetic acid solution. Bacterial cell suspensions (350 µL, 10^6^ CFU/mL) were inoculated into conical tubes containing 6 mL of TSA and 50 µL of the prepared NPs (concentration of the NPs used for these experiments resulted equal to 83 μM). The HDPE coupons were placed in a conical tube and incubated for 48 h at 37 °C. HDPE coupons were placed such that the HDPE could be exposed to both the solution and the air interface. TSA was used as a control. Crystal violet staining was used to visually inspect the biofilm formation and eradication steps. After 48 h of incubation, the HDPE coupons were sequentially removed and washed with phosphate buffer and water. Subsequently, HDPE coupons were separately placed into crystal violet dye solution (2% crystal violet in 95% ethanol) for 5 min. The coupons were washed with water to observe the respective locations of biofilm formation.

### Characterization of *L. parabuchneri* biofilm inhibition using in situ IR-ATR spectroscopy

For the real-time monitoring of *L. parabuchneri* biofilm growth and inhibitions, experiments were performed by the use of a customized horizontal flow cell assembly, which consists of a removable top-plate and a trapezoidal horizontal 45° six-reflection ZnSe crystal with dimensions 72 × 10 × 6 mm [32]. The IR-inactive regions of the ATR element were covered by the spray-coating deposition of AgCl/BAC/TCH/PVMK nanoantimicrobial composite. The identification of IR inactive regions on the ZnSe waveguide was described elsewhere [36]. The active sensing regions along the ATR element were in contact with biofilm formed inside the flow-cell, exposed only to the Ag^+^ ions released by the neighboring regions (3 × 0.5 mm spots of nanoantimicrobials deposition, 0.5 mm thickness) [32]. The IR experiments for the long-term monitoring of biofilms were carried out separately: first, with a ZnSe crystal modified with the AgCl/BAC/TCH/PVMK coating; secondly, with a bare ZnSe waveguide.

The flow-cell setup is connected to the FTIR-ATR multireflection compartment of the Tensor II infrared spectrometer (Bruker Optics, Etlingen, Germany) and a peristaltic pump (Watson Marlow Series 400, Cornwall, UK) with silicon tubing and Luer-lock assemblies. The flow cell was mounted in the multi-reflection compartment sample chamber of the infrared spectrometer. Prior to real-time measurements, the crystal was cleaned in a UV light chamber using intense ultraviolet light. Afterwards, the mounted cells were cleaned with ethanol and rinsed with sterilized water for half an hour. The MRS conditioning film was recorded as the background spectrum for subsequent *L. parabuchneri* biofilms to minimize water interference. The sterilized MRS medium was flushed into the system for 3 h with a delay of 5 min between measurements at a flow rate of 0.7 mL/min, which resulted in a residence time within the flow cell of ~ 150 s. After recording the conditioning film background, the MRS media were replaced with a bacterial solution (OD_600_ = 0.7) for 2 h at a 0.7 mL/min flow rate with a 10 min delay between measurements. This period was optimized on the basis of the required time for initiating the attachment of *L. parabuchneri* biofilms at the ZnSe waveguide [37, 38]. After 2 h of initial attachment of *L. parabuchneri*, sterile MRS medium was pumped again through the FTIR-ATR assembly cell for 24 h at a 0.5 mL/min flow rate. Dynamic flow conditions were necessary to monitor the growth dynamics of *L. parabuchneri* biofilms. Based on the previous studies from D. Bajrami et al., continuous 0.7 mL/min flow rate is optimized for the initial attachment of *L. parabuchneri* biofilms to the ZnSe waveguide [37, 38]. IR signature of biofilms becomes apparent after 2 h from the beginning of the experiment, confirming the initial attachment has occurred. The associated increase of the related IR absorption features related to biofilm constituents such as amides and extracellular polymeric substances are distinct indicators of bacterial adhesion and *L. parabuchneri* biofilm growth [32]. The fresh MRS solution medium washed out the free cells that had not attached and delivered nutrients to the microorganisms. The spectra were recorded at 2 cm^−1^ resolution with 100 averaged spectra in the infrared region of 4000–400 cm^−1^ (1700–989 cm^−1^ region of interest). Data acquisition was performed using Bruker OPUS™ 8.1 software (Bruker, Ettlingen, Germany). Spectra were all converted into Excel datasets using Essential FTIR software (Operant LLC, Madison, WI, USA) and elaborated in the OriginLab software package (OriginLab Corp., Northampton, MA, USA). The IR spectra of the *L. parabuchneri* biofilms were recorded at 22 ± 1 °C in an air-conditioned room. The plots of the integrated peak values (IPVs) were calculated from FTIR spectra based on six spectral regions: amide I (1700–1616 cm^−1^), amide II (1578–1476 cm^−1^), amide III (1350–1200 cm^−1^), nucleic acid (1280–1175 cm^−1^), and EPS (1138–989 cm^−1^) versus time.

## Results and discussion

### Characterization of AgCl/TCH and AgCl/BAC/TCH nanocolloids (NCs)

**Fig. 1:**
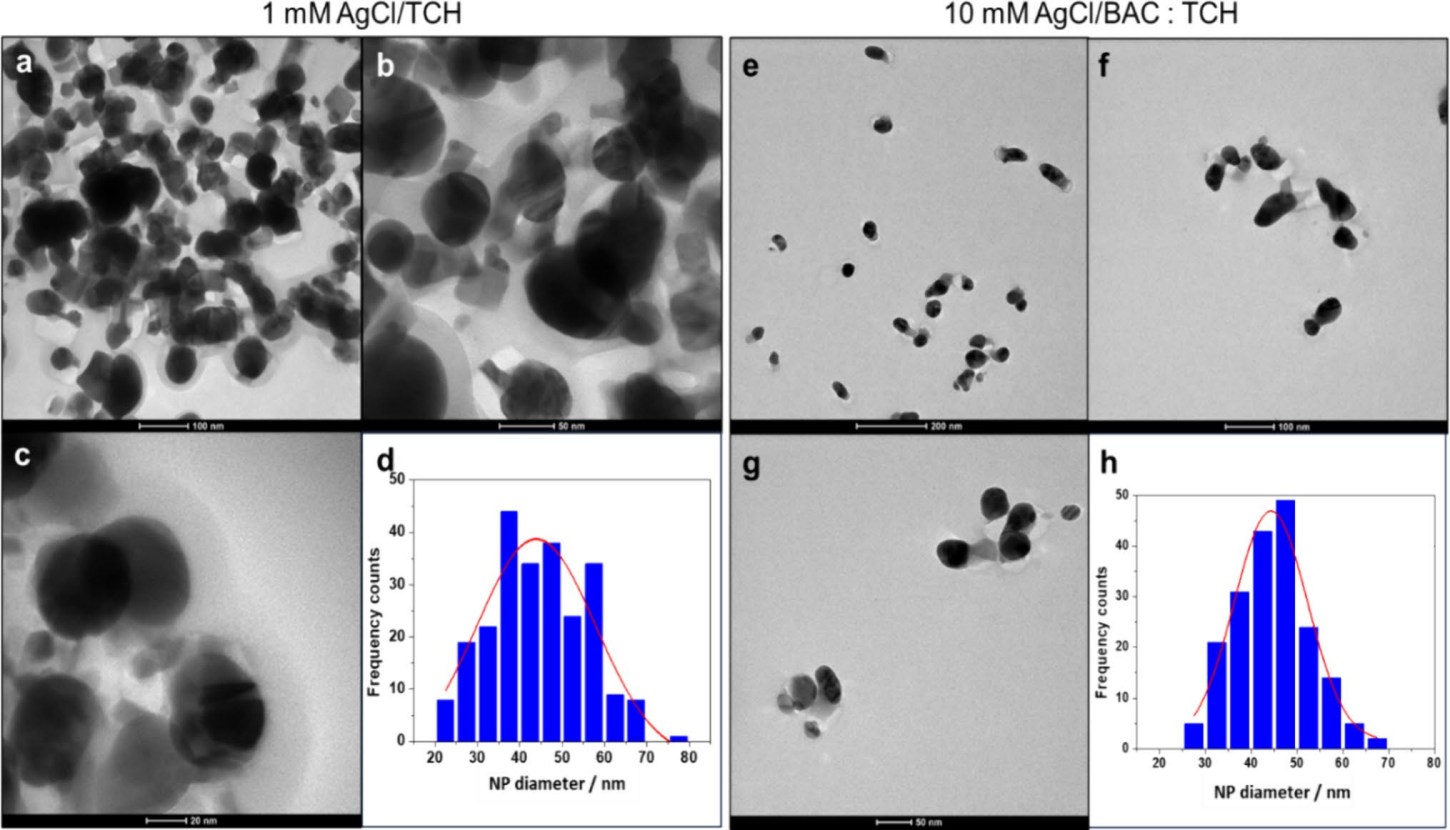
**a**–**c** TEM images and **d** Histogram of 1mM AgCl/TCH. **e**–**g** TEM images and **h** Histogram of 10mM AgCl/BAC/TCH

Initially, TEM characterization was carried out to study the morphology of AgCl NPs. Typical TEM micrographs of 1 mM AgCl/TCH are reported in Fig. 1a–c, which show spheroidal AgCl nanoparticles with an average diameter of 40 ± 15 nm (Fig. 1d). To the best of our knowledge, the formation of AgCl NPs from AgNO_3_ and TCH is a novelty, as the previous literature does not seem to have explored this combination extensively. On the other hand, the formation of NPs is commonly reported in the presence of surfactants [1, 39]. Moreover, TEM shows a grayish layer over individual and aggregated particles, which can be attributed to free TCH molecules in the solution which appeared due to desiccation process of samples before TEM characterization. TEM morphology of 10 mM AgCl/BAC/TCH is shown in Fig. 1e–g, with an average diameter of 45 ± 10 nm AgCl NPs (Fig. 1h), stabilized by BAC molecules. AgCl/BAC/TCH showed improved stability and reduced NPs aggregation compared to AgCl/TCH colloidal system. AgCl NPs size distributions over 20 nm limit nanotoxicity and support their real-life applications.

In view of AgCl/TCH application in real-life products, AgCl/TCH and AgCl/BAC/TCH NPs were subjected to DLS characterization. Furtherly, to evaluate colloidal stability, AgCl/TCH and AgCl/BAC/TCH were diluted with PBS buffer. AgCl/TCH system showed a peak in the volume-weighted size distribution at approximately 1600 nm with a wide size distribution (Fig. S1a) and a -(10 ± 1) mV ζ-potential value. The obtained value indicates an unstable system that is likely to precipitate over time. This instability could be attributed to the presence of a partial amount of free TCH in the solution, as discussed earlier. Buffer-diluted AgCl/TCH showed an average size of approximately 900 nm (Fig. S1b) with a slightly narrower distribution than bare AgCl/TCH, and a highly negative ζ-potential of -(30 ± 5) mV, indicating that the AgCl/TCH are stable in buffer solution possible due to ionic strength effect. The AgCl/BAC/TCH colloid showed an average hydrodynamic diameter of 60 ± 5 nm (Fig. S1c) with a narrow size distribution, and highly positive ζ-potential of 45 ± 2 mV. Buffer diluted AgCl/BAC/TCH showed a bit wider size distribution (Fig. S1d) and a reduced ζ-potential of 20 ± 7 mV if compared to the pristine colloid. When AgCl nanoparticles are dispersed in phosphate buffer, the ζ-potential tends to decrease due to the sorption or complexation of phosphate anions on the surface of the nanoparticles. For citrate-capped AgNPs, it was demonstrated that in the presence of phosphate buffer, the aggregation kinetics were affected by monovalent and divalent electrolytes, leading to changes in critical coagulation concentrations and subsequent NPs behavior [40]. The negative charge on the AgCl/TCH surface does not allow phosphate adsorption, further decreasing the ζ-potential. Conversely, in the case of AgCl/BAC/TCH, the positive surface charge facilitates interaction and complexation, reducing stability in buffer solution, as can be seen by the wider DLS distribution. We are here considering the “rule of thumb” that ζ-potential values higher than ± 30 mV are sufficient to prevent colloid aggregation, and provide information on the long-time stability of our colloidal system [41].

**Fig. 2:**
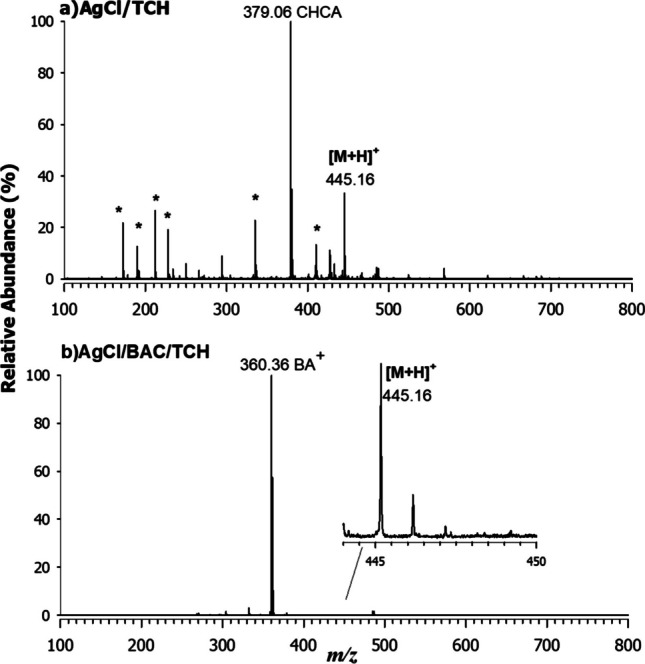
Positive MALDI MS spectra of **a** AgCl/TCH and **b** AgCl/BAC/TCH by using CHCA as a matrix. Interfering matrix-related peaks are labelled with an asterisk

In order to investigate the coordination of AgCl NPs with the TCH molecules, MALDI mass spectrometry characterization was carried out. First, the desorption/ionization efficiency of representative matrices was evaluated by using the aqueous solution of TCH 1 mM. Fig. S2 displays the MALDI MS spectra in positive (a, b) and negative (c) ion mode, using DHB (2,5-di-hydroxy-benzoic acid, a), CHCA (α-cyano-4-hydroxy-cinnamic acid, b), and 9AA (9-Aminoacridine, c) as matrices in the range 100–800 m/z. In all cases, the base peak observed in the spectra is assigned to matrix-related ions, alongside other peaks identified as matrix-dimers, trimers also including sodium and potassium adducts indicated with an asterisk. However, TCH was detected as a protonated adduct ([M + H]^+^) at m/z 445.16 in Fig. S2 (a,b) where M corresponds to the neutral compound. When using DHB, sodiated and potassiated adducts were also generated while with 9AA deprotonated molecule at m/z 443.15 together with a demethylated adduct at *m/z* 427.12 were observed. Interestingly, the lower laser fluence required for CHCA reduces the energy of intermolecular collisions, thus limiting adducts and fragment formation and originating the analyte signal with a good signal-to-noise (S/N) ratio. For these reasons, CHCA was selected for further analyses. To evaluate the global composition of colloidal NPs and the effective antibiotic interaction/stability within the whole system, AgCl/TCH and AgCl/BAC/TCH were pelleted and analyzed by MALDI MS in positive ion mode (Fig. 2).

In the AgCl/TCH NPs the signal of TCH was successfully detected at *m/z* 445.16 as protonated adduct together with already observed interfering matrix-related ions (see figure S2). In AgCl/BAC/TCH NPs, unfortunately, the TCH signal was suppressed by benzyl-dimethyl-hexadecyl-ammonium ion [C_25_H_46_N]^+^ at m/z 360.36, which efficiently ionized due to the permanent charge of quaternary ammonium (note a small fragment due to the loss of two methyl groups at m/z 332.33). The TCH peak at m/z 445.16 was indeed present but at very low intensity (see inset in the Fig. 2b). To confirm the correct attribution of the peak related to TCH, MS/MS spectra were collected on the isolated ion at m/z 445.16 and compared to tandem mass spectrum of the standard antibiotic (Fig. S3). At the end, the MALDI characterization allowed us to conclude that the TCH was efficiently included in the investigated colloidal systems.

### Antimicrobial activity of AgCl/TCH and AgCl/BAC/TCH NCs

**Fig. 3:**
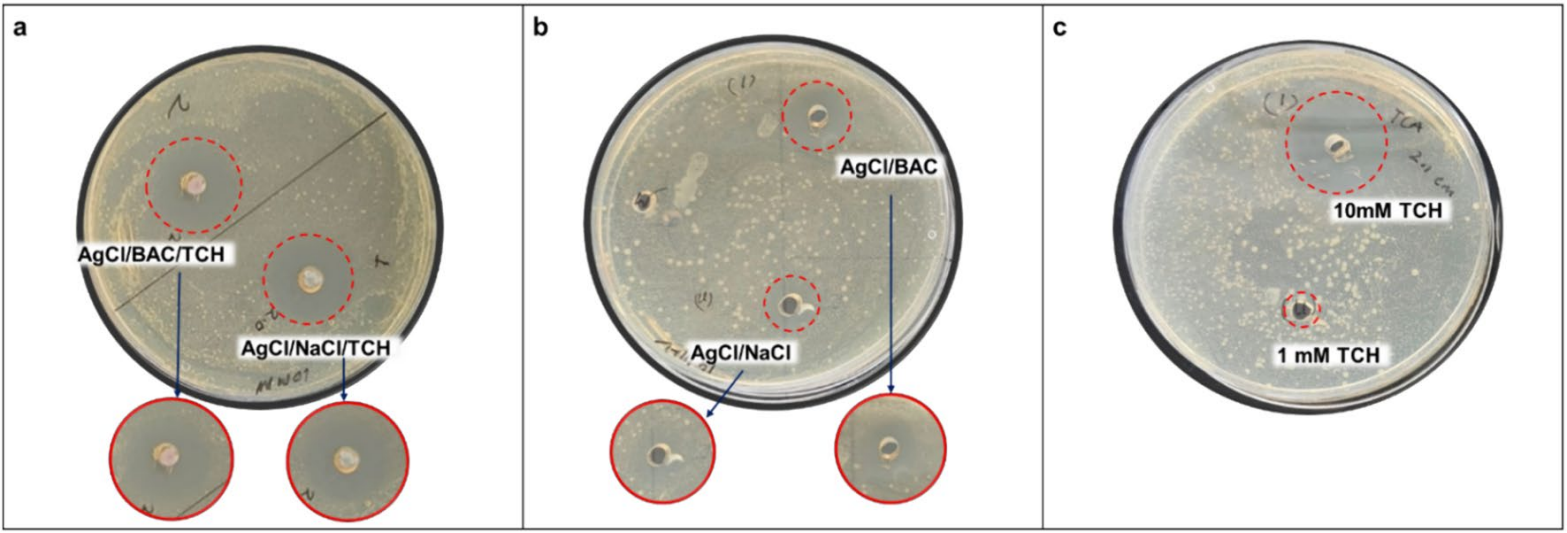
Antimicrobial susceptibility well diffusion assay: **a** 10 mM AgCl/BAC/TCH and 10 mM AgCl/NaCl/TCH; **b** 10 mM AgCl/BAC and 10 mM AgCl/NaCl, and **c** 10 mM TCH and 1 mM TCH against *S. enterica* on tryptic soy Agar (TSA)

Preliminarily, the well-known conventional Agar well diffusion method was used to evaluate the antimicrobial properties of all the colloidal systems. Samples containing AgCl and NaCl were prepared as positive controls in absence of either BAC or TCH, to keep the amount of Cl^−^ counterions constant in all the samples. The following systems were tested against *S. enterica*: 10 mM AgCl/BAC/TCH 10 mM AgCl/NaCl/TCH, 10 mM AgCl/BAC, 10 mM AgCl/NaCl, 10 mM TCH, and 1 mM TCH (Fig. 3). Addition of 1 mM TCH into 10 mM AgCl/BAC and AgCl/NaCl nanocolloids allowed reaching an inhibition zone diameter of 18.0 ± 1.5 mm (10 mM AgCl/BAC:TCH) and 17.0 ± 1.5 mm (AgCl/NaCl:TCH), respectively. Without the inclusion of 1 mM TCH, 10 mM AgCl/BAC, and AgCl/NaCl NPs showed inhibition zone diameters of 11 ± 2 mm and 8 ± 2 mm, respectively. As expected, higher activity was observed for 10 mM TCH (19 ± 2 mm) than for 1 mM TCH (6 ± 2 mm) and 1 mM AgCl/TCH (7 ± 2 mm). All results are summarized in Table 1. It is worth underlining that the 10 mM AgCl/BAC/TCH system exhibited almost the same activity as 10 mM TCH, and a clearly higher antimicrobial activity than 10 mM AgCl/BAC and 10 mM AgCl/NaCl NPs. Additionally, either 1 mM AgCl/TCH or 1 mM TCH were not enough to exert sufficient antimicrobial activity; on the contrary, when 1 mM TCH was included in the 10 mM AgCl/BAC/TCH system, the antimicrobial efficacy reached the level of the 10 mM TCH system. This phenomenon can be attributed to the antibiotic addition in the system, which synergistically improves the antimicrobial performance of both AgCl/BAC and AgCl/NaCl NPs. The AgCl/BAC/TCH system is preferred over AgCl/NaCl/TCH because BAC moieties are able to provide sufficient protection to AgCl from photodegradation, and improve their stability over time [1, 16].

**Table 1:** Summary of zone of inhibition diameters of NPs and TCH antibiotics

Samples	Zone of inhibition (mm)
10 mM AgCl/BAC/TCH	18.0 ± 1.5
10 mM AgCl/NaCl/TCH	17.0 ± 1.5
10 mM AgCl/BAC	11 ± 2
10 mM AgCl/NaCl	8 ± 2
10 mM TCH	19 ± 2
1 mM AgCl/TCH	7 ± 2
1 mM TCH	6 ± 2

The viability reduction of the foodborne pathogenic bacteria *S. enterica* in the means of the optical density values (OD = 600 nm) was evaluated for the AgCl/BAC/TCH nanocolloidal solutions added in the bacterial suspensions. The reduction in vitality, indicated by the OD values, was observed when comparing *S. enterica* as the positive control (OD_600_ = 0.75) to the TCH antibiotic alone (OD600 = 0.15) and surfactant BAC (OD_600_ = 0.7) with synthesized AgCl/BAC nanocolloids (OD_600_ = 0.24). Notably, the highest vitality reduction was observed with the synergistic system AgCl/BAC/TCH (OD_600_ = 0.06) significantly proving the super nonantimicrobial nature of such system in the direction of total inhibition of bacterial growth. Figure S4 displays CFU counting plates illustrating the colonies corresponding to viability reduction against the bacterial strain *S. enterica*. Tab. S1 shows the CFU counting values of the bacterial strain after treatment with AgCl/BAC/TCH, AgCl/BAC, TCH and BAC compared to *S. enterica*, without antimicrobial treatment. The CFU/mL was used to quantify the viable cells and showed a total inhibition activity of inoculum detected after treatment with AgCl/BAC/TCH compound for each bacterial strain. The distinguished low number of CFU counts for the TCH itself, highlight its pivotal role in precisely quantifying viable cells, reflecting the potent antimicrobial activity of the synergistic AgCl/BAC/TCH system. The absence of colonies observed for Gram-negative *S. enterica* after treatment with AgCl/BAC/TCH nanocolloidal solution indicates no viable cells, affirming the effectiveness of the antimicrobial treatment.

The minimum inhibition concentration MIC value we found for TCH antibiotic alone (against *S. enterica*) is compatible with literature one, and equal to 5 μg/mL [42]. The minimum inhibitory concentration (MIC) of AgCl/BAC against the Gram-negative bacterium *S. enterica* CECT4594 strain was determined to be 8 μg/mL. However, the addition of TCH to the AgCl/BAC formulation resulted in increased efficiency, with the lowest MIC value observed at 1.4 μg/mL against the same strain. In contrast, susceptibility tests against the Gram-positive bacterium *L. parabuchneri* DSMZ 5987 revealed a MIC value of 13 μg/mL for AgCl/BAC. Remarkably, the combination of AgCl/BAC/TCH exhibited the lowest MIC value overall, at 1.7 μg/mL. The standard control test involving the surfactant alone, conducted for both strains, yielded an MIC value of 256 μg/mL, representing the minimum inhibitory concentration of BAC.

### Antibiofilm activity of AgCl/BAC/TCH NCs

**Fig. 4:**
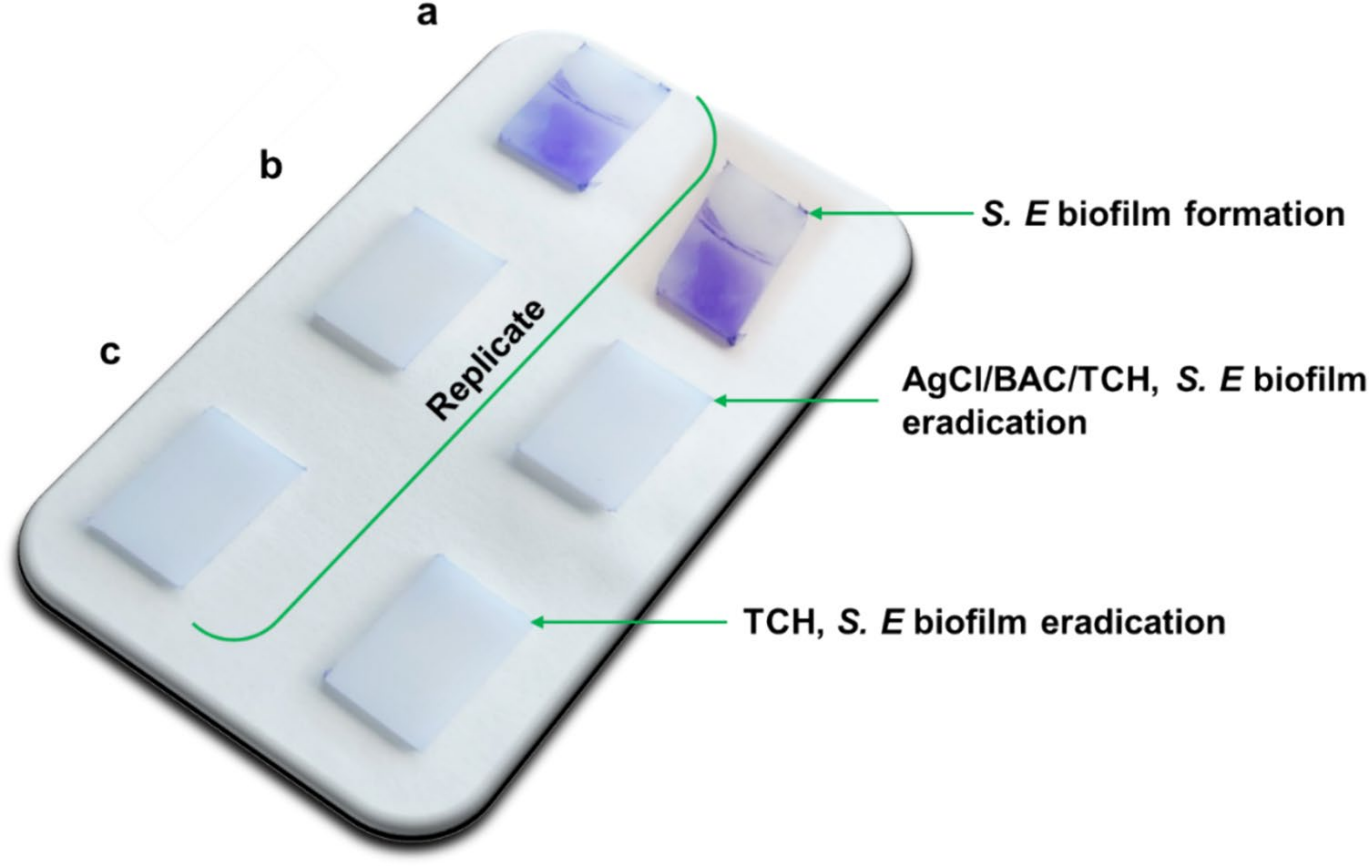
HDPE coupons into *S. enterica* bacterial suspension: biofilm formation (**a**), biofilm eradication by 10 mM AgCl/BAC/TCH (**b**), and 10 mM TCH (**c**)

The industrial biofilm investigation protocol was performed using 10 mM AgCl/BAC/TCH NPs and 10 mM TCH for the formation of foodborne pathogenic *S. enterica* biofilms. Figure 4a shows biofilm detection and formation without the involvement of any NPs or antibiotics; Antibiofilm activity was evaluated following crystal violet staining protocol (CV assay) applied on the high density polyethylene (HDPE) surfaces. The stained areas indicated the biofilm presence, while the areas which stayed clear verified the absence of its growth [37, 43]. In the present study, it appears that without antimicrobial NPs in the bacterial suspension, *S. enterica* (*S. E.*) bacteria formed both areal and strong interfacial biofilms on the HDPE coupon. In this case, bacteria grew significantly in the liquid medium (turbid solution, data not shown); hence, they tended to attach to the HDPE surface. In regard to the addition of either 10 mM AgCl/BAC/TCH NPs or 10 mM TCH antibiotic in the bacterial suspension, the effects of Ag^+^ ions, BAC surfactant, and antibiotic molecules are combined, thus inhibiting sufficiently bacterial growth in the liquid medium, and resulting in inhibited biofilm formation at the HDPE surface (Fig. 4b). This result is comparable to the activity of 10 mM TCH antibiotic on HDPE coupons (Fig. 4c).

### In situ IR-ATR biofilm inhibition study by AgCl/BAC/TCH/PVMK coating

The combined use of nanoantimicrobials and antibiotics is a robust emerging strategy to reduce microbial infections and to potentiate the high antimicrobial activity of such nano-formulated systems [44, 45]. The novel idea to use traditional antibiotics such as tetracycline hydrochloride (TCH) in combination with AgCl/BAC nanoantimicrobials is a great alternative over conventional antibiotics to overcome biofilm-associated AMR with low toxicity to human health [46]. In this section, the study aimed at screening molecular changes in bacterial biofilms influenced by the combination of BAC and TCH for anti-biofilm activity against *L. parabuchneri* biofilms.

**Fig. 5:**
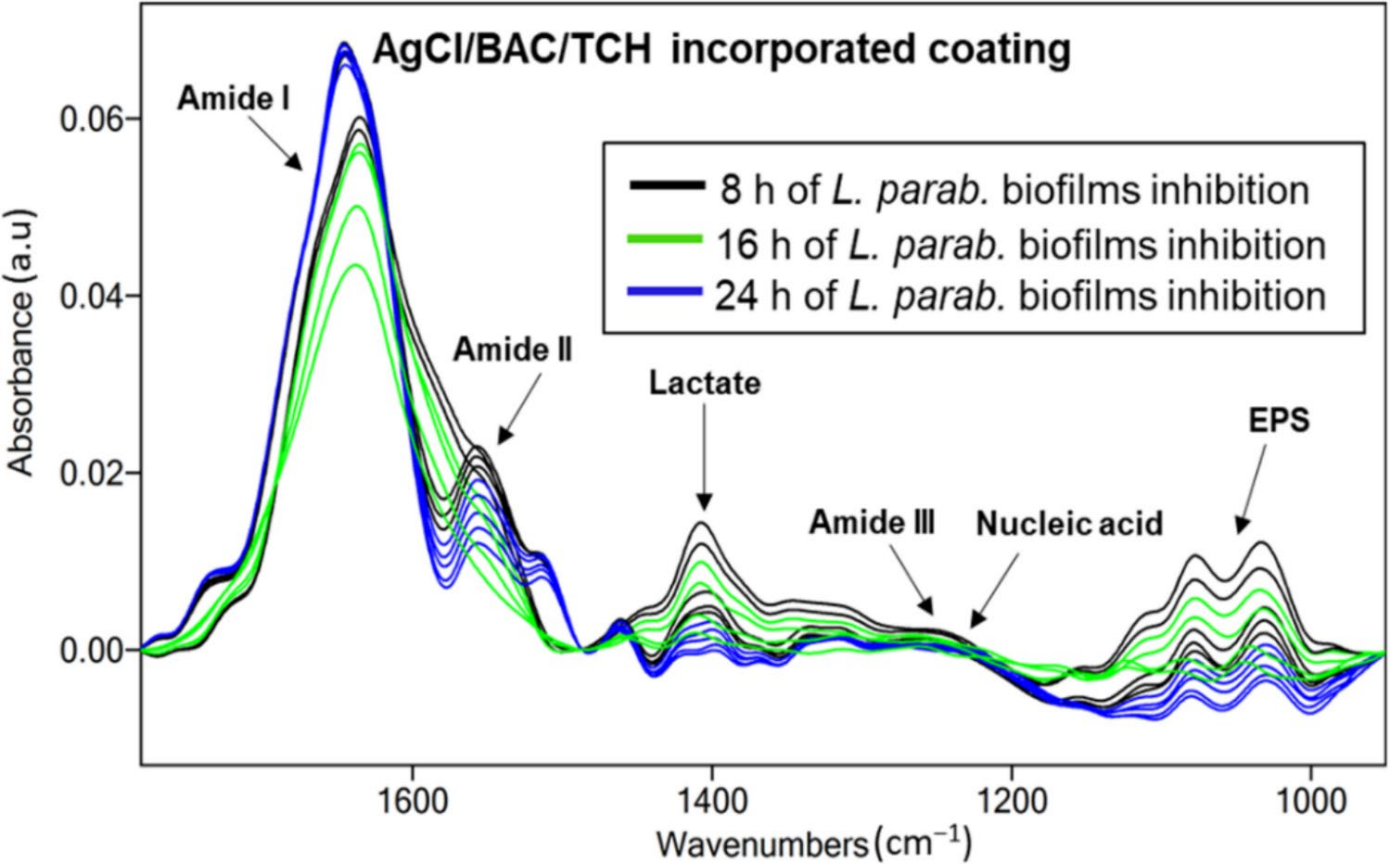
Temporal evolution of relevant IR bands for *L. parabuchneri* biofilm inhibition on the waveguide modified by AgCl/BAC/TCH coating in inactive sensing regions. Infrared ATR spectra of 8 h of biofilm inhibition (black lines); 16 h of biofilm inhibition (green lines); and 24 h (blue line) of *L. parabuchneri*. Signal attributions are highlighted by arrows

IR-ATR spectroscopy is a versatile analytical method for studying microbial biofilms in real-time conditions at molecular level [38, 47, 48]. In situ IR studies of biofilm formation and inhibition on the surfaces of both bare and AgCl/BAC/TCH/PVMK-modified ZnSe crystals provided an overview of the fundamental mechanisms involved in biofilm attachment and disruption. A suspension of *L. parabuchneri* DSMZ5987 in the stationary growth phase in sterile MRS media was used for the biofilm formation experiment on bare and patterned ZnSe crystals. During the inoculum of microbial suspension into the flow system, the accumulation at the ZnSe crystal surface and the increase of the microbial coverage was evident through the increase of the assigned infrared bands related to the absorption features of the *L. parabuchneri* biofilms [37]. Spectra were recorded at 10-min intervals under continuous flow conditions (0.7 mL/min flow rate of peristaltic pump) for periods up to 24 h. All the vibrational bands revealed signature of the characteristic constituents for the microbial cell wall membrane, ribosomes, nucleotides, capsule, peptidoglycan [38]. The continuous increase of the biofilm biomass [32] for the blank experiment in absence of antimicrobial agents is compared to the results obtained on the AgCl/BAC/TCH/PVMK-modified ZnSe crystal. The spectra obtained as a function of time for *L. parabuchneri* biofilm inhibition on the waveguide modified by AgCl/BAC/TCH/PVMK coating are shown in Fig. 5. A comparison between the spectra after 8, 16, and 24 h of biofilm monitoring with their corresponding band attributions is observed.

**Fig. 6:**
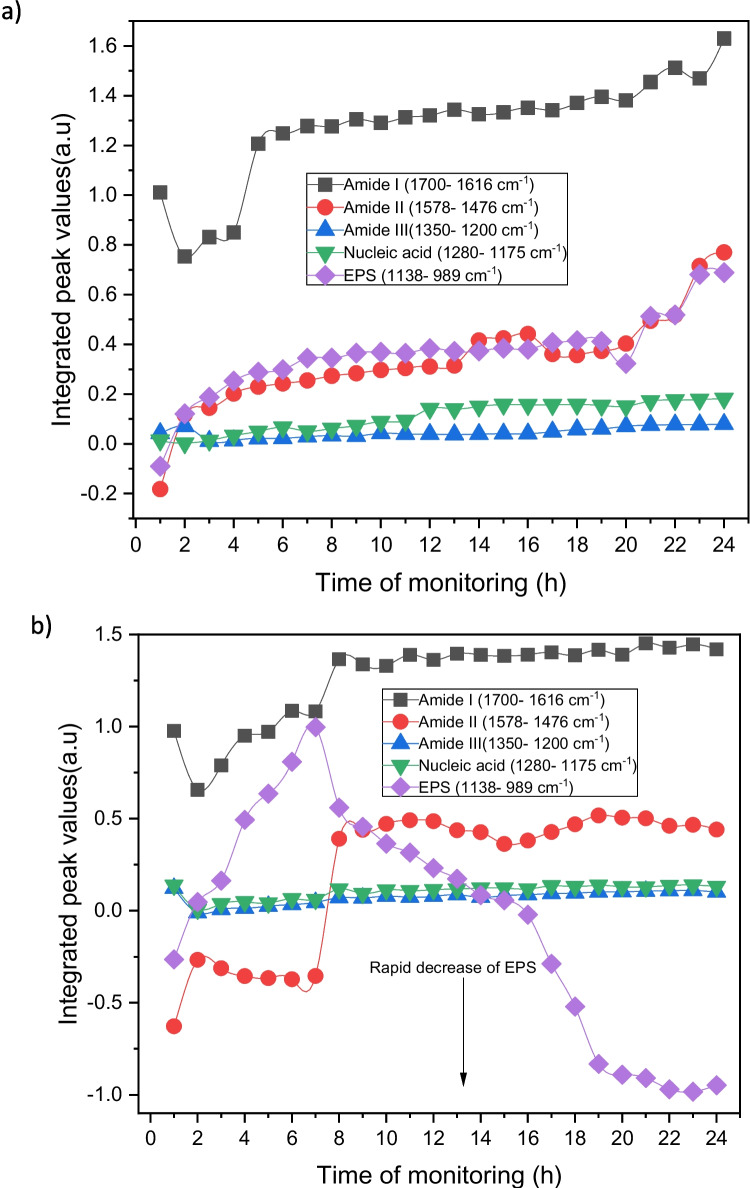
Integrated peak values (IPVs) as a function of time for L. *parabuchneri* biofilm formation for 24 h of monitoring **a** on bare crystal and *L. parabuchneri* biofilm growth inhibition for 24 h of monitoring **b** on top of the modified crystal. The arrow indicates a decrease in the IR bands associated with the EPS content

By plotting integrated peak values (IPVs) as a function of time for the five spectral areas of infrared bands associated to amide I (1700–1616 cm^−1^), amide II (1578–1476 cm^−1^), lactate production (1465–1293 cm^−1^), amide III (1350–1200 cm^−1^), nucleic acid (1280–1175 cm^−1^), and extracellular polymeric substance (EPS) (1038–989 cm^−1^), it is possible to monitor the molecular changes indicating microbial adhesion and biofilm growth [32]. Using the pristine ZnSe crystal, during the initial biofilm growth stages, the constant accumulation of biomass at the bare waveguide surface was apparent, and an increase in bacterial coverage was associated to the increase in IPV values for the corresponding vibrational bands (Fig. 6a). The levels of amide I tended to increase rapidly after 5 h of biofilm monitoring. It is evident that EPS and amide II levels significantly increased as a function of time, showing similar IPVs values. The inflation of the amide II band is characteristic of bacterial colonization of the crystal surface [49]. After approximately 4 h, the amount of EPS and amide II appeared to be reduced, increasing the surface coverage; therefore, the unoccupied areas of the waveguide surface could be considered negligible. The level of nucleic acid/amide III band remained almost constant after first 2 h, suggesting that amide bond in membrane proteins and fatty acid chains gave rise to prominent bands [50]. Accordingly, fewer nucleic acids were synthesized compared to lipopolysaccharides during the attachment of bacteria for 24 h. Despite this, the nucleic acid content started to slightly increase after 12 h.

The same experiment was repeated on the AgCl/BAC/TCH/PVMK-modified ATR crystals (Fig. 6b). In this case, the nonantimicrobial coating was carefully deposited only on IR-inactive regions of the waveguide. The examined biofilm spots were exposed only to Ag^+^ species released by neighboring regions, without direct contact with antimicrobial nanoparticles (NPs). The inhibition of *L. parabuchneri* biofilm growth by the AgCl composite film demonstrates a biostatic action [32], as Ag^+^ released by the coating penetrate the cell membrane wall, leading to disruption of the cell structure and disorder in its organization. Therefore, a lower surface coverage was visible on the ZnSe waveguide surface, and the process of biofilm growth was delayed over time. The presence of the antimicrobial coating will induce stress accompanied with a hinder of *L. parabuchneri* biofilm growth through cellular membrane perturbation [51]. The amide I band at 1645 cm^−1^, (υ_C=O_ coupled with υ_N-H_), is relevant to an increase in membrane lipids and fatty acid contents in microbial cells [52]. A sharp decrease of the amide II band at 1547 cm^−1^ instead, is associated with the removal of biofilm portions from the colonized ZnSe surface [53]. Surprisingly, the steady increase of the amide I and amide II band and the slight shift after the first 8 h, confirm that the recolonization of some spots along the ZnSe crystal may be occurred. The prominent IPVs decrease in the EPS band is due to the gradual antifouling effect of the AgCl/BAC/TCH/PVMK coating (Fig. 6b). The level of the nucleic acid/amide III band dropped in the first 2 h and then remained constant for the whole monitored period.

The extracellular polymeric matrix (EPS) consists of different components, such as glycogen, phosphodiester, phosphorylated proteins, phospholipids, and polyphosphate products [54]. The symmetric stretching vibrations of carbohydrates (υ_sC–OH_, υ_sC–O_) at 1124 cm^−1^ and symmetric stretching vibrations of phosphates (${\upsilon s}_{{\text{PO}}_{2}^{-}}$) at 1083 cm^−1^, seen in Fig. 5, are spectral changes responsible for peptidoglycan, nucleoid and lipopolysaccharides in general, as the main cellular components of *L. parabuchneri* biofilms [32, 55, 56]. Under real-time conditions, it was observed that the band υ_P=O_ at 1645 cm^−1^, related to EPS content, dropped drastically after 8 h and reached zero within approximately 14 h (Fig. 6b). Symmetric stretching vibrations of phosphoryl groups around 990 cm^−1^ (υ_sC–C_, υ_sP–O–P_), identified as the functional constituent of ribosome skeleton (ARN), are related to metabolic changes inside the biofilms [38]. This indicates a complete biofilm eradication from the ZnSe surface, as the EPS is considered the main molecular component responsible for bio-adhesion onto surfaces [57, 58]. The biostatic effect of the AgCl/BAC/TCH/PVMK nonantimicrobial coating, issued by BAC as a well-known disinfecting and stabilizing material in combination with TCH antibiotic, serves as the basis for a complete eradication of food-contaminant *L. parabuchneri* biofilms. Thus, the reduced presence of intercellular components is directly associated with the disruption of *L. parabuchneri* bacterial membranes and partial inhibition of biofilm proliferation. The formulation of such synergistic nanoantimicrobials potentially limits the possibility of antibiotic resistance, thereby effectively hindering microbial biofilms.

## Conclusions

We developed a scalable, green, facile, and fast route to produce antimicrobial and antibiofilm materials combining bioactive AgCl nanoparticles, a renowned disinfecting agent, such as benzalkonium chloride, and a conventional antibiotic molecule, i.e. tetracycline. Conventional agar well diffusion antimicrobial test and industrial biofilms investigation protocol support the fact that 10 mM AgCl/BAC/TCH NPs can be considered potential agents for the inhibition or eradication of food pathogenic *S. enterica* sessile bacteria and biofilms. Moreover, *L. parabuchneri* microbial biofilm growth inhibition was monitored via in situ flow FTIR-ATR spectroscopy, which supported the potentiality of the proposed system. The proposed mild and active AgCl NPs, with sizes greater than 20 nm, might overcome nanotoxicity issues. The addition of biosafe and bioactive BAC further improved NP stability and exerted synergistic antimicrobial and antibiofilm effects. Notably, the inclusion of TCH antibiotic remarkably enhanced the antimicrobial and antibiofilm activities of such green system. This work reveals that 9:1 ratio of BAC and TCH containing AgCl NPs brings improved antimicrobial and antibiofilm activity. These phenomena draw a potential route which outline the necessity to drastically lower antibiotic addition into metal NPs. The prepared hybrid antibacterial system could improve synergistic bacteriostatic and bactericidal action, provide reduction in therapeutic doses, overcome nanotoxicity and dose related toxicity issues, reduce treatment duration, and fight antimicrobial resistance.

### Supplementary Information

Additional file1 (DOCX 464 kb)

## Data Availability

Not applicable.
