# Supplementary material for: Development of super nanoantimicrobials combining AgCl, tetracycline and benzalkonium chloride
Source: Discov Nano. 2024 Jun 11;19(1):100. doi: 10.1186/s11671-024-04043-3 (PMC11166621; doi:10.1186/s11671-024-04043-3)
Supplement: Supplementary file 1 — Additional file1 (DOCX 464 kb) [file 11671_2024_4043_MOESM1_ESM.docx]

**Discover Nano**

**Supplementary Information**

**Development of super nanoantimicrobials combining AgCl, Tetracycline and Benzalkonium Chloride**

Syed Imdadul Hossain^a,b,1^, Diellza Bajrami^c,1^, Nazan Altun^d^, Margherita Izzi^a,b^, Cosima Damiana Calvano^a,b^, Maria Chiara Sportelli^a,b^, Luigi Gentile^a,b^, Rosaria Anna Picca^a,b^, Pelayo Gonzalez^d^, Boris Mizaikoff***^c,e^ and Nicola Cioffi***^a,b^

^a^ Chemistry Department, University of Bari Aldo Moro, Via E. Orabona, 4, 70126 Bari, Italy

^b^ CSGI (Center for Colloid and Surface Science) c/o Dept. Chemistry, Via E. Orabona, 4, 70126 Bari, Italy

^c^ Institute of Analytical and Bioanalytical Chemistry, Ulm University, Albert Einstein-Allee 11, 89081 Ulm, Germany

^d^ ASINCAR (Research Association of Meat Industries of Principado de Asturias), 33180 Noreña, Spain

^e^ Hahn-Schickard, Sedanstrasse 14, 89077 Ulm, Germany

^1^ These authors contributed equally.

*Corresponding authors. B. M: [boris.mizaikoff@uni-ulm.de](file:///C:\Users\rosan\Documents\Picca_Dell\Hossain\AgCl_DDAC_DDoAC\boris.mizaikoff@uni-ulm.de); N. C: [nicola.cioffi@uniba.it](file:///C:\Users\rosan\Documents\Picca_Dell\Hossain\AgCl_DDAC_DDoAC\nicola.cioffi@uniba.it)

**Determination of vitality of cells by CFU counting**

Bacterial vitality reduction was examined for AgCl/BAC/TCH, AgCl/BAC, BAC and TCH materials. 50 µL of each antimicrobial product were added in 350 µL from 2^nd^ dilution of bacterial culture of S. enterica (10^6^ CFU/mL) and then as a mixture added in 6 mL TSA. The tubes were incubated at 37°C for 48 h. Subsequently, any cells that did not adhere were eliminated by rinsing the coupon twice with 2 mL of PBS buffer. The mixture was thoroughly vortexed and then subjected to serial dilutions at 1:10^4^ – 1:10^8^. The quantity of bacteria adhered was assessed by plating 100 μL of each serial dilution on TSA agar plates and then incubated at 37°C for 24 h [1]. Each strain and material underwent three replicates, utilizing separate cultures for each experiment. Results were presented as Log 10 CFU (mean ± SD of the replicates).


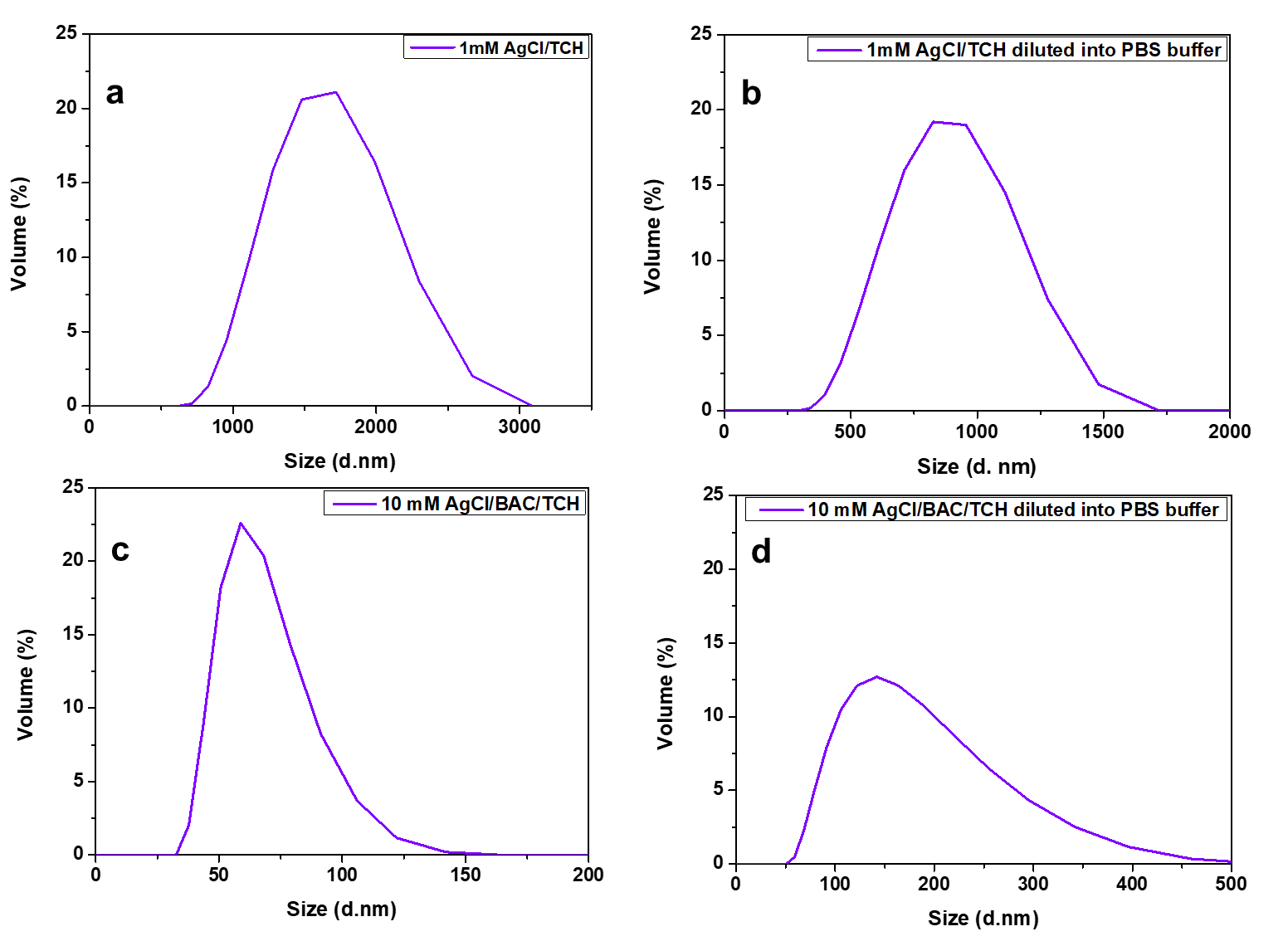


**Fig. S1** Size distribution by volume % of (a) 1mM AgCl/TCH, (b) 1mM AgCl/TCH diluted into PBS buffer, (c) 10 mM AgCl/BAC/TCH, (d) 10 mM AgCl/BAC/TCH diluted into PBS buffer





**Fig. S2** (a, b) Positive MALDI MS spectra of a standard TCH solution (1 mM) using (a) DHB and (b) CHCA as matrices. (c) Negative MALDI spectrum using 9AA as a matrix. Interfering matrix-related peaks are labelled with an asterisk. The structure of tetracycline is reported in the inset





**Fig. S3** Positive MALDI MS/MS spectra of the ion at *m/z* 455.16 in (a) AgCl/TCH, (b) AgCl/BAC/TCA and (c) TCH standard solution (1 mM) using CHCA as a matrix. TCH is characterized by two diagnostic ions at *m/z* 154 and *m/z* 410 (Boscher A. et al., Jo Chrom A. 2010;1217(41):6394–6404. doi: 10.1016/j.chroma.2010.08.024)


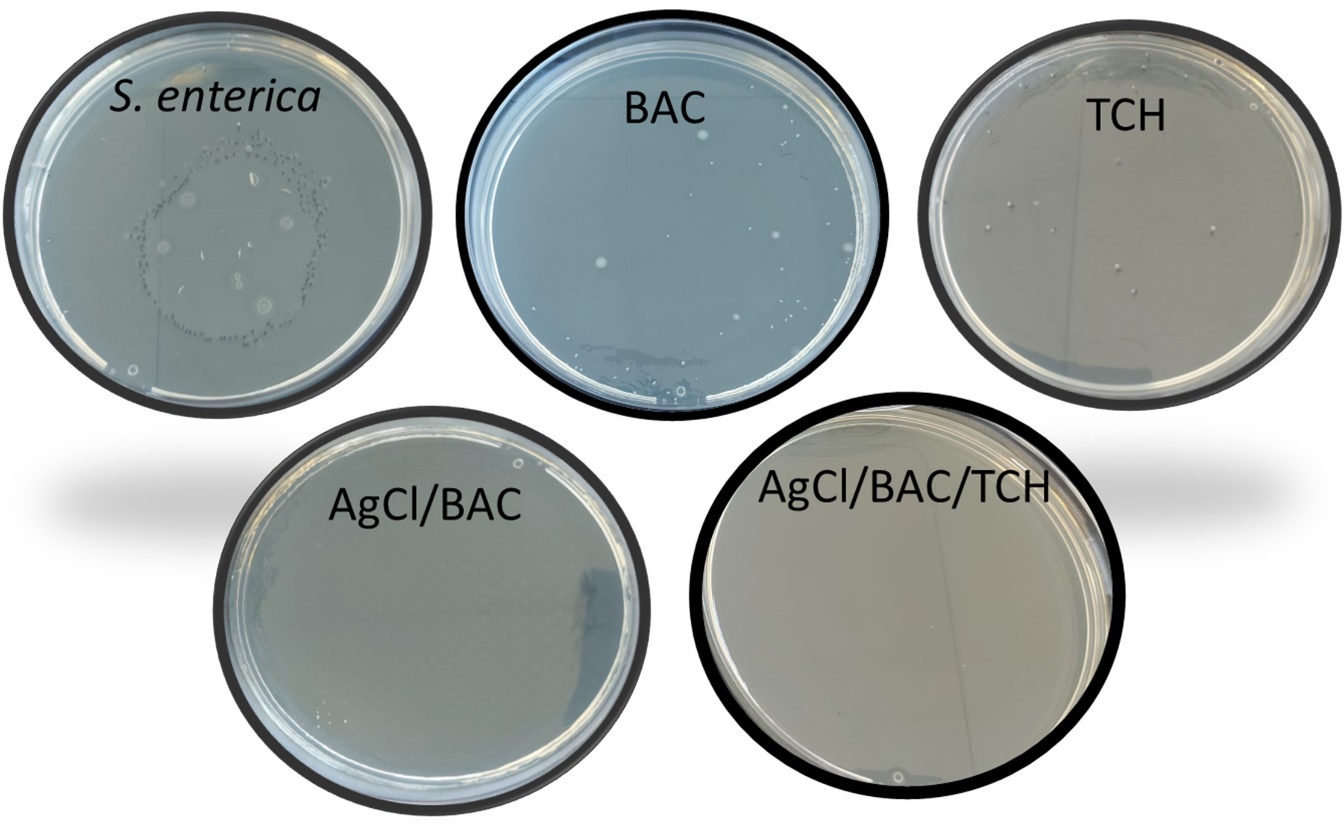


**Fig S4** Colony forming unit (CFU) counting studies for the viability reduction of synthesized nanoantimicrobials observed in the cultured plates for the *S. enterica*.

**Table S1**. Colony-forming unit (CFU) counts of *S. enterica* for the serially diluted 10 folds of cultured suspension with nanocolloidal solutions of developed nanoantimicrobials. The values are presented as the mean of three replicates ± standard deviation (SD).

| Colony forming unit (CFU/mL) ± SD | | | | | | |
| --- | --- | --- | --- | --- | --- | --- |
| Bacteria | + Control | BAC | TCH | | AgCl/BAC | AgCl/BAC/TCH |
| *S. enterica* CECT4594 | 6.23E^+09^± 1 | 2.6E^+07^± 1 | | 36 ± 2 | 1558 ± 2 | 0 ± 1 |

**Table S2.** The antimicrobial results of AgCl/TCH and AgCl/BAC/TCH are compared to those obtained with similar AgCl based materials.

| Antimicrobial agent | Bacterial inhibition | Strains tested | References |
| --- | --- | --- | --- |
|  |  |  |  |
| AgCl/BAC/TCH | Diameter of inhibitory zones 18.0 ± 1.5 mm, 1.4-1.7 μg/mL (MICs) | *Salmonella enterica*  *Lentilactobacillus parabuchneri* | Present Study |
|  |  |  |  |
| AgCl/BAC | Diameter of inhibitory zones 11 ± 2 mm, 8-13 μg/mL | *Salmonella enterica*  *Lentilactobacillus parabuchneri* | Present Study |
| AgCl/DDAC | Diameter of inhibitory zones 13–26 mm for 2-16 μg/mL (MICs) | *E. coli* ATCC 25922  *L. monocytogenes* 46  *S. aureus* ATCC 29213  *P. aeruginosa* ATCC 27853 | [2] |
| AgCl/DDoAC | Diameter of inhibitory zones 15–23 mm for 16-128 μg/mL (MICs) | *E. coli* ATCC 25922  *L. monocytogenes* 46  *S. aureus* ATCC 29213  *P. aeruginosa* ATCC 27853 | [2] |
| Biosynthesized Ag/AgCl NPs using *Chara* algae extract | Diameter of inhibitory zones up to 18.7 mm, 13–68μg/mL (MICs) | *Staphylococcus aureus*,  *Escherichia coli*,  *Klebsiella pneumonia*, and  *Pseudomonas aeruginosa* | [3] |
| AgCl NPs based on LDH, LDHa and LDHb | 2.8–8.5 μg/mL (MICs) | *S. aureus (ATCC 29213),*  *S. epidermidis (ATCC 12228),*  *P. aeruginosa (ATCC 15692) and one fungal species Candida albicans (CAF2/1)* | [4] |
| Ag/AgCl NPs synthesized from *Fusarium oxysporum* | Diameter of inhibitory zones up to 6.0 mm, 10.52 μg/mL (MIC) | *Serratia mascescens, Klebsiella pneumoniae carbapenemase-KPC strains and E. coli 25922* | [5] |

**References:**

[1] N. Altun, M.F. Hervello, F. Lombó, P. González, Using staining as reference for spectral imaging: Its application for the development of an analytical method to predict the presence of bacterial biofilms, Talanta 261 (2023) 124655. https://doi.org/10.1016/j.talanta.2023.124655.

[2] D. Bajrami, S.I. Hossain, A. Barbarossa, M.C. Sportelli, R.A. Picca, L. Gentile, F. Mastrolonardo, A. Rosato, A. Carocci, N.A. Colabufo, B. Mizaikoff, N. Cioffi, A scalable route to quaternary ammonium-functionalized AgCl colloidal antimicrobials inhibiting food pathogenic bacteria and biofilms, Heliyon 10 (2024) e25260. https://doi.org/10.1016/j.heliyon.2024.e25260.

[3] K.T. Hassan, I.J. Ibraheem, O.M. Hassan, A.S. Obaid, H.H. Ali, T.A. Salih, M.S. Kadhim, Facile green synthesis of Ag/AgCl nanoparticles derived from Chara algae extract and evaluating their antibacterial activity and synergistic effect with antibiotics, Journal of Environmental Chemical Engineering 9 (2021) 105359. https://doi.org/10.1016/j.jece.2021.105359.

[4] M. Nocchetti, A. Donnadio, V. Ambrogi, P. Andreani, M. Bastianini, D. Pietrella, L. Latterini, Ag/AgCl nanoparticle decorated layered double hydroxides: synthesis, characterization and antimicrobial properties, J. Mater. Chem. B 1 (2013) 2383–2393. https://doi.org/10.1039/C3TB00561E.

[5] S. Picoli, M. Durán, P. Andrade, N. Duran, Silver nanoparticles/silver chloride (Ag/AgCl) synthesized from Fusarium oxysporum acting against Klebsiella pneumouniae carbapenemase (KPC) and extended spectrum beta-lactamase (ESBL), Frontiers in Nanoscience and Nanotechnology 2 (2016) 107–110. https://doi.org/10.15761/FNN.1000117.
